# Major Gaps in Understanding Dietary Supplement Use in Health and Disease

**DOI:** 10.1146/annurev-nutr-011923-020327

**Published:** 2023-05-17

**Authors:** Regan L. Bailey, Shinyoung Jun, Alexandra E. Cowan, Heather A. Eicher-Miller, Jaime J. Gahche, Johanna T. Dwyer, Terryl J. Hartman, Diane C. Mitchell, Rebecca A. Seguin-Fowler, Raymond J. Carroll, Janet A. Tooze

**Affiliations:** 1 Institute for Advancing Health Through Agriculture, Texas A&M University, College Station, Texas, USA; 2 Department of Cancer Biomedical Science, National Cancer Center Graduate School of Cancer Science and Policy, Goyang-si, Republic of Korea; 3 Department of Nutrition Science, Purdue University, West Lafayette, Indiana, USA; 4 Office of Dietary Supplements, National Institutes of Health, Bethesda, Maryland, USA; 5 Jean Mayer US Department of Agriculture Human Nutrition Research Center on Aging, Tufts University, Boston, Massachusetts, USA; 6 Department of Epidemiology, Rollins School of Public Health, Emory University, Atlanta, Georgia, USA; 7 Department of Statistics, Texas A&M University, College Station, Texas, USA; 8 Department of Biostatistics and Data Science, Wake Forest School of Medicine, Winston-Salem, North Carolina, USA

**Keywords:** dietary supplement, validity, reproducibility, dietary assessment, measurement error, methodology

## Abstract

Precise dietary assessment is critical for accurate exposure classification in nutritional research, typically aimed at understanding how diet relates to health. Dietary supplement (DS) use is widespread and represents a considerable source of nutrients. However, few studies have compared the best methods to measure DSs. Our literature review on the relative validity and reproducibility of DS instruments in the United States [e.g., product inventories, questionnaires, and 24-h dietary recalls (24HR)] identified five studies that examined validity (*n* = 5) and/or reproducibility (*n* = 4). No gold standard reference method exists for validating DS use; thus, each study’s investigators chose the reference instrument used to measure validity. Self-administered questionnaires agreed well with 24HR and inventory methods when comparing the prevalence of commonly used DSs. The inventory method captured nutrient amounts more accurately than the other methods. Reproducibility (over 3 months to 2.4 years) of prevalence of use estimates on the questionnaires was acceptable for common DSs. Given the limited body of research on measurement error in DS assessment, only tentative conclusions on these DS instruments can be drawn at present. Further research is critical to advancing knowledge in DS assessment for research and monitoring purposes.

## INTRODUCTION

**Figure 1:**
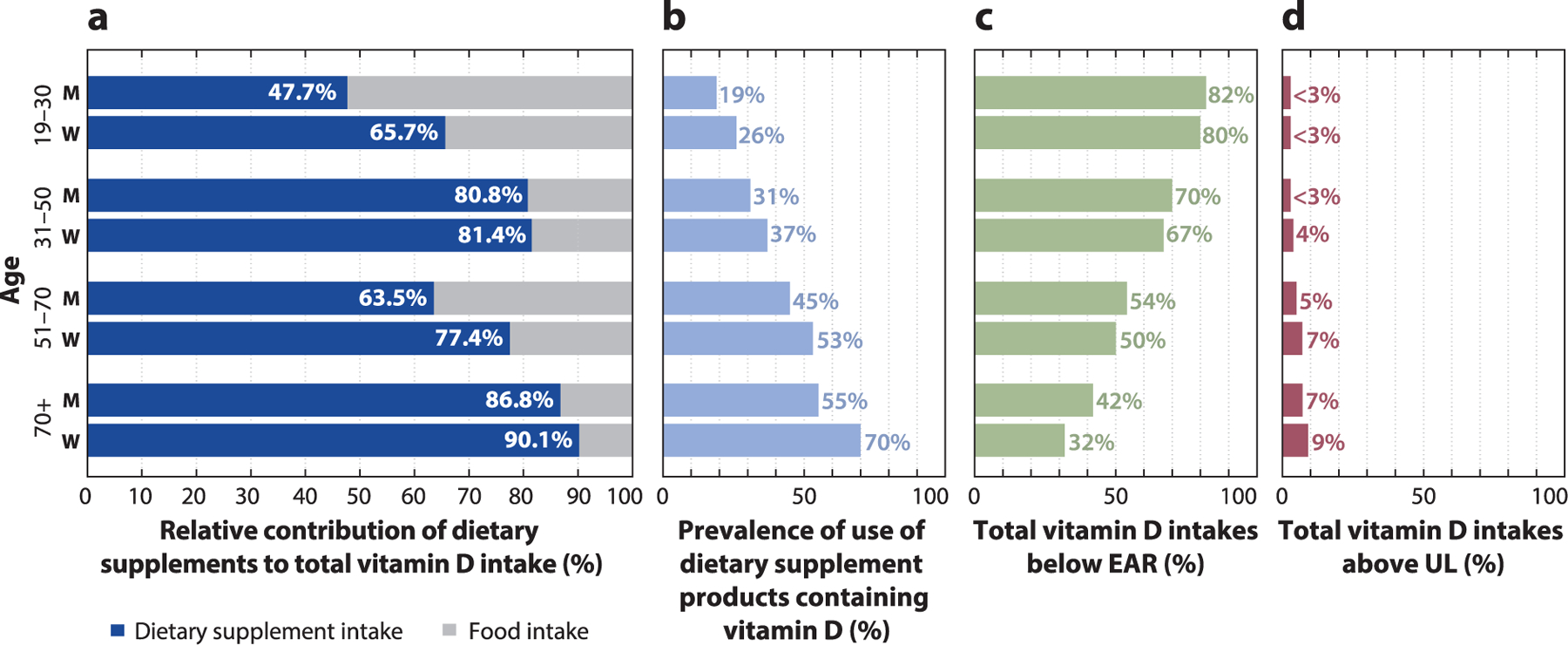
Total vitamin D intake among US adults by sex and age. Subjects were ≥19 years old, were not pregnant or lactating, and had complete dietary information for the day 1 and day 2 24-h dietary recalls. (*a*) The percentage within each bar reflects the relative contribution of dietary supplements to total usual micronutrient intake of vitamin D. (*b*) The percentage for each bar reflects the prevalence of use of dietary supplement products containing vitamin D among the respective population subgroup. (*c*) The percentage for each bar reflects the estimated proportion of the population with total usual vitamin D intakes below the EAR among the respective population subgroup. (*d*) The percentage for each bar reflects the estimated proportion of the population with total usual vitamin D intakes above the UL among the respective population subgroup. Data from Reference 60. Abbreviations: EAR, estimated average requirement; M, men; UL, tolerable upper intake level; W, women.

**Figure 2:**
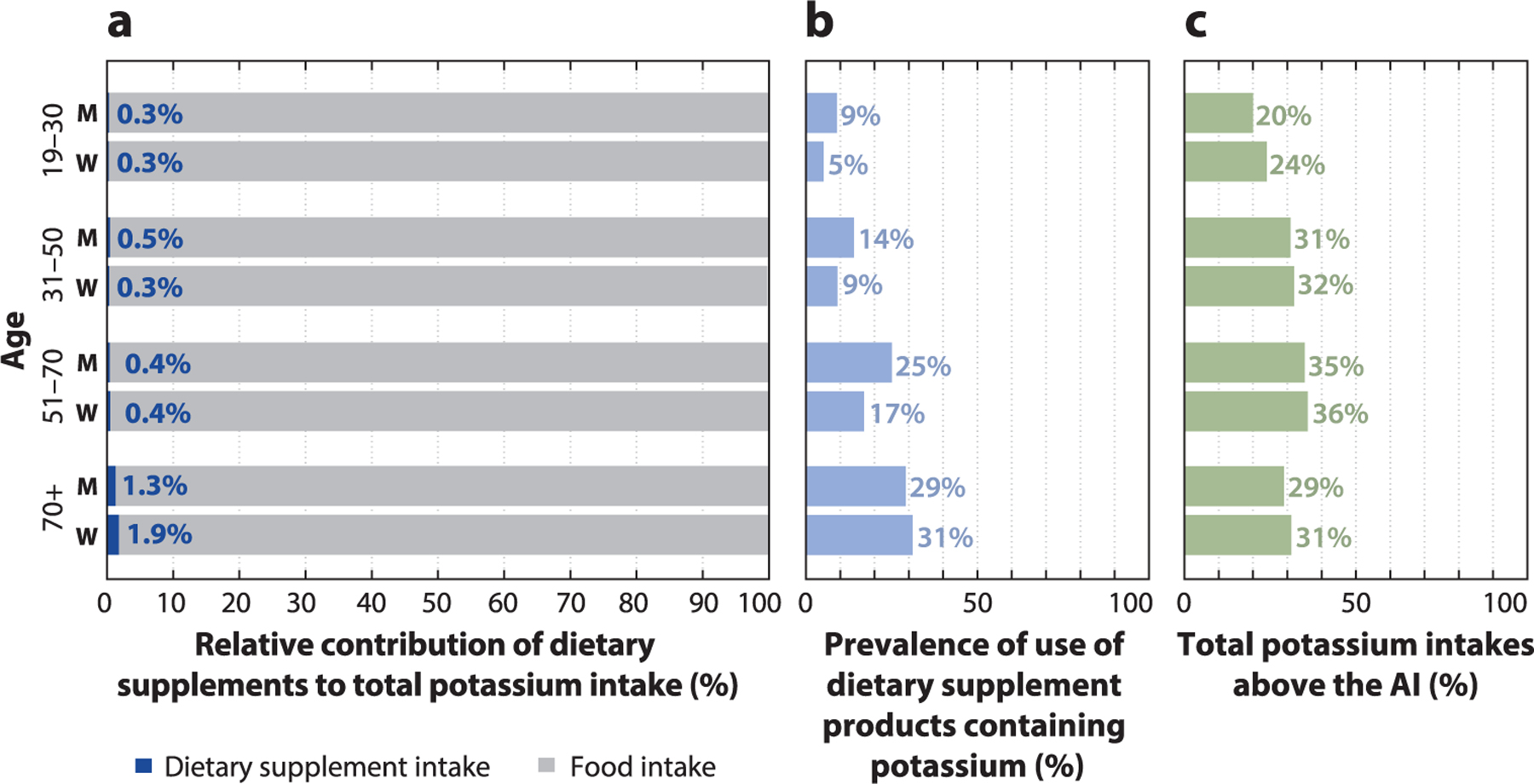
Total potassium intake among US adults by sex and age. Subjects were ≥19 years old, were not pregnant or lactating, and had complete dietary information for the day 1 and day 2 24-h dietary recalls. (*a*) The percentage within each bar reflects the relative contribution of dietary supplements to total usual micronutrient intake of potassium. (*b*) The percentage for each bar reflects the prevalence of use of dietary supplement products containing potassium among the respective population subgroup. (*c*) The percentage for each bar reflects the estimated proportion of the population with total usual potassium intakes above the AI among the respective population subgroup. An AI is established when sufficient scientific evidence is not available to establish an EAR. The proportion of the population exceeding the UL for potassium was not evaluated due to low population prevalence of excessive potassium intake. Data from Reference 60. Abbreviations: AI, adequate intake; EAR, estimated average requirement; M, men; UL, tolerable upper intake level; W, women.

Poor nutrient intakes are an important and modifiable risk factor for multiple chronic diseases of public health concern. Nutrition research has provided invaluable insights into the role that diet plays on health and chronic disease risk; however, the focus of these studies has traditionally relied on nutrient intakes from foods alone. Currently, more than half of US adults and one-third of US children use at least one dietary supplement (DS) product (38, 56). DS use is even more widespread among certain population groups such as women (32), cancer survivors (20, 22, 36, 37, 46, 52, 61, 62), older adults (26, 32), pregnant and lactating women (9, 13, 31, 48), and individuals with a higher family income or living with food security (14, 30). Moreover, many DSs are formulated with large doses of several vitamins and minerals (4), such as multivitamin-mineral (MVM) DSs, which are the most frequently used products and typically provide 100% or more of the recommended daily amounts for several micronutrients (12). For example, nationally representative survey data among US adults suggest that DSs provide an estimated 77% (or greater) of total vitamin D intake among certain population subgroups, reducing the proportion of the population at risk of inadequate vitamin D intake, as well as increasing the proportion of the population at risk of potential excess (Figure 1). Nearly 7% of older (70+ years) men and 9% of older women were at risk of excessive intake of vitamin D in 2015–2018 (60) (Figure 1). However, for nutrients less commonly found in DSs, such as potassium, nearly all intake is estimated to be obtained from the diet (i.e., foods and beverages), with DS intake contributing *<*2% of total potassium intake among US adults (60) (Figure 2). These findings lend support to the idea that despite variations in the contribution of DSs to total intake by nutrient, estimates of total micronutrient exposures inclusive of intakes from all sources (i.e., foods/beverages and DSs) have the potential to reduce the proportion of the population classified as at risk of inadequate dietary intakes and increase the proportion that has intakes considered to be potentially excessive (5, 6). Thus, to accurately estimate the prevalence of potential inadequacy and excess, it is important that not only nutrient exposures from foods and beverages but also nutrient exposures from DSs be included in research studies aimed at understanding health outcomes or risk of disease in order to ensure the validity of diet–health associations (4). However, the most appropriate methods to assess the prevalence of DS use and the contribution of DSs to nutrient exposures are largely unknown.

DS use has been assessed using methods that only consider intake from DSs, such as frequency-based questionnaires, screening tools, and DS product inventories, as well as using methods that measure DS exposures in combination with exposures from foods and beverages, including 24-h dietary recalls (24HRs), food frequency questionnaires (FFQs), and food records/diaries (3). While decades of work have been devoted to improving the validity and reproducibility of food and beverage intake assessment, only limited efforts have been made with DSs, and few studies have compared multiple DS instruments to one another. Nicastro and colleagues (43) found that the estimated prevalence of MVM use is lower on the 24HR alone than on the DS inventory in the National Health and Nutrition Examination Survey (NHANES), suggesting that methodological differences may exist between the 24HR and the inventory methods for estimating the prevalence of use of MVM DSs; Cowan et al. (15) also demonstrated that the prevalence estimates for overall DS use, as well as the amounts of nutrients consumed from DSs, vary depending on the method of DS assessment in NHANES. Unique to NHANES, during the 24HR, the interviewer specifically inquires about any products taken by the participant and reported during the in-home inventory (43); therefore, the 24HR is not independent from the inventory method in NHANES, suggesting that further research examining independent DS assessment methods is necessary. Moreover, little is known about the accuracy, reliability, and measurement error structure of DS assessment methods, all of which are likely to differ from those of foods in several ways (e.g., energy under-reporting, errors in estimating portion size, and social desirability, among others) (4). Thus, the purpose of this systematic review is to examine the existing literature on the validity and reproducibility of each of the DS assessment methods that are independent of each other and currently utilized in the US, given that DSs are defined and regulated differently around the world (2, 21). For this review, the validity of a dietary assessment method is defined as the “extent to which a test measure of a concept agrees with a reference measure” (28, p. 410). Note that no reference measure exists for DS assessment, and validity should be interpreted as relative validity. The reproducibility of a measure (i.e., test-retest reliability) was taken to reflect the extent to which two repeated measures can produce the same result after repeat assessments (34).

## METHODS

### Eligibility Criteria

Studies were selected for inclusion if their primary objective was to evaluate the relative validity and/or reproducibility of a DS assessment tool and if they focused predominantly on independent DS assessment method comparisons. Thus, descriptive analyses that estimated the prevalence of DS use or nutrient intakes from DSs but did not include a validity or reproducibility component were not admitted to the review. Studies were excluded if they were not original research (e.g., review articles), were conducted in countries other than the United States, were published in any language other than English, and/or were not full-text articles (e.g., abstracts and conference proceedings). It is also important to note that there were no exclusion criteria based on participant characteristics.

### Literature Search Strategy

This review is registered in the PROSPERO International Prospective Register of Systematic Reviews (CRD42021281023; https://www.crd.york.ac.uk/prospero).

A search of four electronic databases (PubMed, Google Scholar, Scopus, and EBSCO) was conducted from August 1 to September 1, 2021, on studies published between January 1, 1990, and September 1, 2021. Search terms, used alone or in combination, included terms related to DS products (dietary, supplement, vitamin, mineral, multivitamin, and nutritional), terms related to assessment methods (24HR, record, recall, food frequency questionnaire, questionnaire, method, assessment, dietary, instrument, inventory, and history), and terms related to the validation and reliability of DS assessment methods (relative validity, validation, reproducibility, reliability, feasibility, usability, and calibration). Secondary searches of the reference lists of all identified articles were also conducted manually. The authors of the articles were not contacted if any information was missing. A systematic approach to the review of the literature was taken; however, given the limited number of articles identified in the data extraction process (described in further detail below), this review should be considered more narrative in nature.

**Figure 3:**
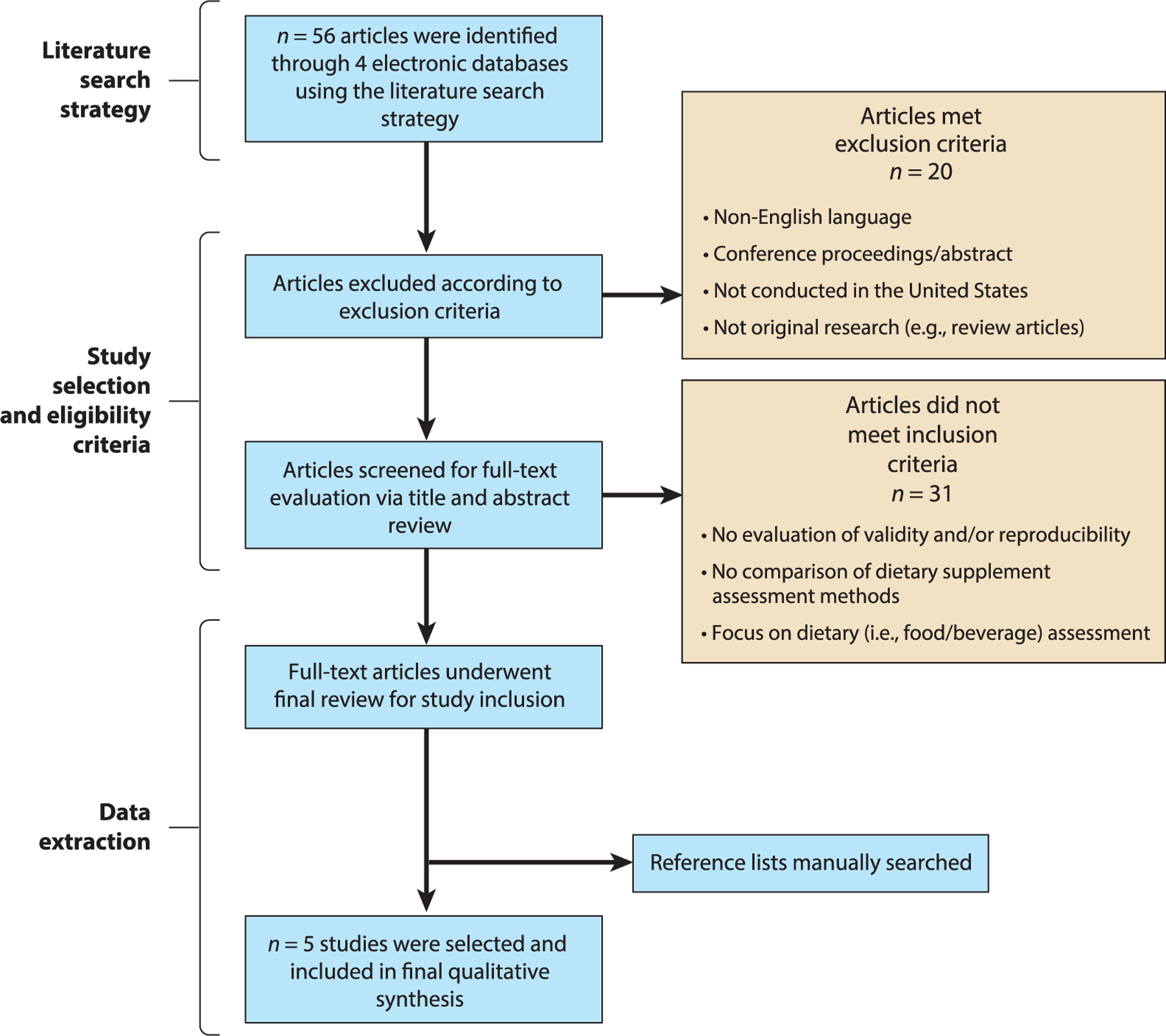
Flow chart of studies for inclusion in the systematic review of the validity and reproducibility of dietary supplement assessment methods in the United States.

The flow chart of studies for inclusion at each step of the review process is provided in Figure 3. The initial search of the databases yielded 56 articles based on the search terminology. Of the identified articles, 20 were deemed ineligible based on exclusion criteria. Titles and abstracts of the remaining articles were then screened for full-text retrieval according to the inclusion criteria (i.e., validity or reproducibility of the study and comparison of DS assessment methods); for any article in which it was unclear whether the study met the inclusion and/or exclusion criteria after title and abstract screening, the full text of the article was retrieved and evaluated. An additional 31 articles were omitted at this step, resulting in 5 remaining articles. These 5 articles underwent final review for study inclusion by two independent reviewers (S.J. and R.L.B.), and any disagreements regarding study inclusion were resolved via discussion with a third independent party (A.E.C.). An additional review was also conducted by an experienced systematic reviewer (i.e., a science librarian). Data from each of the 5 identified articles were extracted by two of the investigators into an Excel^®^ spreadsheet and categorized according to whether the assessment method or methods’ validity, reproducibility, or both were evaluated.

### Methods of Statistical Evaluation of Validity and Reproducibility

The studies presented herein vary in terms of the methods used to evaluate the validity and reproducibility of DS assessment methods. A kappa statistic (κ) is a measure of agreement or the proportion of agreement beyond that expected by chance (35). In general, κ or weighted κ methods were the most commonly employed statistics for categorical outcomes (e.g., user/nonuser and frequency of use). For the purposes of this review, the κ was interpreted according to standards used in previous literature as follows: values ≤0 indicating no agreement, 0.01–0.20 indicating slight agreement, 0.21–0.40 indicating fair agreement, 0.41–0.60 indicating moderate agreement, 0.61–0.80 indicating substantial agreement, and 0.81–1.00 indicating almost perfect agreement (35).

Spearman’s and/or Pearson’s correlation coefficients were commonly used for continuous outcomes (e.g., mean or median amounts of nutrients). Pearson’s and Spearman’s correlation coefficients quantify the strength of the linear relationship between estimates; correlations *<*0.3 were considered to be negligible; 0.3–0.5, low; *>*0.5–0.7, moderate; *>*0.7–0.9, high; and *>*0.9, very high, based on previous literature (39). Two studies (29, 53) also utilized intraclass correlation coefficients (ICCs) to evaluate test-retest reliability between repeat measures (e.g., first and second administrations of a questionnaire), which is another measure often used to quantify the consistency between two DS assessments, with the difference being that this statistical approach is considered a measure of agreement rather than just association (i.e., measuring the same construct rather than just related constructs) and typically assumes a pooled mean and variance. ICCs were interpreted in an identical fashion to Pearson’s and Spearman’s correlation coefficients. One study examined the proportion of agreement for categorical outcomes via the percent of classification matches between two instruments (55). This study (55) also evaluated the reproducibility of amounts of nutrients reported on repeat administrations of a self-administered questionnaire by evaluating differences in log-transformed supplemental nutrient intakes reported on both supplement frequency questionnaire (SFQ) administrations (55; see also “Example 38.9: Testing Equality of Covariance and Correlation Matrices” in 51).

## RESULTS

### Overview

The search identified five studies evaluating the relative validity (29, 40, 45, 53, 55) and/or reproducibility (29, 40, 53, 55) of DS assessment methods in the US population (Table 1). All studies were observational by design, and most (*n* = 4) were conducted among a subsample of a large cohort. All five studies recruited only US adults (age range at recruitment: 21 to 75 years) and included both men and women. Two of the five studies recruited mostly non-Hispanic White and highly educated participants (*>*90%) (45, 53), whereas the other three studies included participants from diverse ethnic backgrounds, including non-Hispanic Black (i.e., African American), Hispanic and Latino, Japanese American, and Native Hawaiian Americans (29, 40, 55). Two studies reported data from the state of Washington (45, 53), two studies reported data from California and Hawaii (40, 55), and one study reported data from 35 states (29).

For relative validity, all five studies utilized a self-administered, frequency-based DS questionnaire as an assessment tool relative to a reference instrument of choice (29, 40, 45, 53, 55). In addition to a self-administered questionnaire, one study (45) also assessed the relative validity of a telephone interview. In terms of reference instruments, three (45, 53, 55) of the five studies used a DS product inventory and label transcription approach, although the details of inventory administration varied (e.g., in-home versus at-clinic, photocopy versus manual label transcription). Two studies compared the assessments to 24HRs (29, 40), and one study also compared assessment methods for specific nutrients to nutritional biomarkers (53). For the four studies (29, 40, 53, 55) that assessed reproducibility, all utilized repeat administrations of self-administered questionnaires to evaluate consistency between estimates. Details on the design and participant characteristics of the studies evaluating relative validity are provided in Supplemental Table 1 and reproducibility in Supplemental Table 2.

### Studies Conducted in Washington State

Patterson et al. (45) compared the validity of an in-person, detailed interview with DS product label transcription (i.e., via photocopy of supplement bottles) to (*a*) a telephone interview that queried vitamin and mineral use and (*b*) a self-administered DS questionnaire [similar to the National Cancer Institute/Block Health Habits Questionnaire (11)] among a sample of adult DS users (*n* = 104; mean age 44 years) (45). The authors sought to collect the same information on the dose, duration, and frequency of common multivitamin and single-nutrient DS products via the use of each instrument, but the methods differed in their mode of administration (i.e., interviewer-versus self-administered) and format (open- versus closed-ended) (45). When assessing agreement between the reported prevalence of DS product types in the telephone interview and the in-person interview/label transcription methods, agreements (via κ) ranged from κ = 0.14 for other multivitamins [i.e., all other mixtures of vitamins and/or minerals that are not a once-a-day type of multivitamin with or without minerals (e.g., antioxidants or calcium/magnesium/zinc)] to κ = 0.92 for multivitamins (i.e., a once-a-day type of multivitamin with or without minerals) (45). On the self-administered DS questionnaire, somewhat different patterns were observed, with agreements ranging from κ = 0.36 for calcium to κ = 0.70 for vitamin E, showing fair to substantial agreement across all DS product types examined (45) (Supplemental Table 1). Spearman’s correlation coefficients were used to compare (*a*) average daily DS intakes for the telephone interview to the criterion measure and (*b*) the self-administered questionnaire to the criterion measure. Correlations for the telephone interview ranged from *r* = 0.27 (iron) to *r* = 0.75 (vitamin C), whereas, for the self-administered questionnaire, correlations ranged from *r* = 0.08 (iron) to *r* = 0.76 (vitamin C) when compared with an in-person interview/label transcription (45) (Supplemental Table 1).

Satia-Abouta and colleagues (53) examined the validity of a comprehensive self-administered DS questionnaire that collected information on vitamin and mineral use relative to an in-person interview with manual label transcription of the supplement bottles by trained interviewers, among a cohort of US adults who participated in the Vitamins and Lifestyle (VITAL) study (*n* = 149; participants were Washington state residents with high educational attainment) (53). A self-administered questionnaire was designed for this study to collect detailed information on 10 vitamins and 6 minerals from different product types over the previous decade, administered at baseline and at 3 months (53). The criterion measure to examine validity, similar to that of Patterson et al. (45), was an in-person participant interview with label transcription; Pearson’s correlations comparing current supplemental nutrient intakes for 16 nutrients reported on the two instruments ranged from 0.58 (beta-carotene) to 0.82 (chromium) among users of DSs (53) (Supplemental Table 1). Nutritional biomarkers (serum beta-carotene, serum alpha-tocopherol, plasma vitamin C, and spot urinary calcium) were also employed as a reference method and were correlated with quartiles of nutrient intakes in the randomly selected validation sample (*n* = 149) and in this sample plus 77 super users of DSs with high DS doses reported (adjusted for age, sex, race, smoking status, and body mass index) (53). Correlations were considered moderate for vitamin E (*r* = 0.69), low for vitamins A (*r* = 0.31) and C (*r* = 0.29), and negligible for calcium (*r* = −0.07) for intakes reported on the questionnaire but were presumably higher for intakes reported on the inventory method (Supplemental Table 1). Reproducibility of estimates reported on the questionnaire administered at baseline and at 3 months was assessed via ICCs, with correlations ranging from 0.69 (beta-carotene) to 0.87 (vitamin E), with a mean of 0.79 (53) (Supplemental Table 2). Weighted κ was also used to assess consistency between questionnaire administrations and resulted in slightly lower agreement, spanning from κ = 0.58 (calcium) to κ = 0.78 (multivitamins) and a mean of κ = 0.69 (Supplemental Table 2).

### Hawaii–Los Angeles Multiethnic Cohort Study

Murphy and colleagues (40) collected three 24HRs and compared DS reporting with a self-administered questionnaire (administered twice, ~2.4 years apart) on the prevalence and frequency of DS use, doses, and product types for eight vitamin and mineral DSs over the previous year among *n* = 2,377 adults (45–75 years old). Frequency of DS use reported on the questionnaire and the average of the 24HRs among DS users and nonusers and across six DS use categories (e.g., 0 to ≥3 DS/day) were assessed via κ and weighted κ statistics, with κ ranging from 0.17 (vitamin A) to 0.72 (vitamin E) among DS users and nonusers; nearly identical findings were noted across the six frequency-of-use categories (Supplemental Table 1). When comparing nutrient amounts from DSs reported on the two instruments, intakes tended to be higher on the questionnaire than the average of the 24HRs for most nutrients examined (40) and resulted in Pearson’s correlations that varied appreciably by nutrient, from *r* = 0.11 for folate to *r* = 0.71 for vitamin E (Supplemental Table 1). Reproducibility of the questionnaire with administrations approximately 2.4 years apart ranged from fair to substantial, with the greatest amount of agreement between questionnaires for vitamin C (κ = 0.64), followed by vitamin E and calcium (both κ = 0.58), multivitamin DSs (κ = 0.56), beta-carotene and selenium (both κ = 0.46), iron (κ = 0.43), and vitamin A (κ = 0.39) (40) (Supplemental Table 2).

The Multiethnic Cohort Supplement Reporting (MEC-SURE) study is considered the most rigorous and comprehensive comparison of DS assessment methods (25) because detailed information was collected in up to five in-home DS inventories with pill counts, label photos, and a database created specifically for this study. Steffen and colleagues (55) sought to examine the accuracy of a brief, self-administered DS frequency questionnaire, known as the SFQ, relative to the comprehensive inventory among older adults who participated in the MEC-SURE (*n* = 1,029; mean age 68 years) study (55). MEC-SURE participants were randomly categorized into the inventory (described above) or control group; both groups completed the SFQ at baseline and at the 1-year follow-up (55). The SFQ probed individuals on their weekly use of any vitamins or minerals in the previous year, as well as information on frequency, dose, and duration of DS use for multivitamins (e.g., one-a-day, B-complex, and antioxidants), 11 single vitamins and minerals (beta-carotene; calcium; folic acid; iron; zinc; and vitamins A, C, D, and E), fish oil/omega-3s, and garlic DSs (55). Nutrients from DSs (using information on frequency and dose) were then summed as total amounts across all DS product types to obtain daily nutrient intakes (55). The comparison measure, the comprehensive DS inventory, was conducted by a trained interviewer in the home and collected DS information from the product containers (e.g., dose and serving size) for participants in the inventory group (55). Daily, quarterly, and annual nutrient intakes were approximated by summing the nutrient amounts (i.e., frequency and dosage information) across all DSs for each time frame (55). The agreement between the self-administered SFQ and the comprehensive DS inventory methods for the prevalence of weekly DS use was assessed among those in the inventory group by evaluating the percent of classification matches between the two instruments (55). Steffen et al. (55) found that agreement for the prevalence of DS use was generally quite high (≥88%) for all nutrients except vitamin D (74%) (Supplemental Table 1).

In addition to agreement, Steffen et al. (55) also evaluated supplemental nutrient intakes (as geometric means) reported via each method as a measure of relative validity (Supplemental Table 1). At the individual level, the SFQ significantly underestimated mean amounts of vitamin D and folate and overestimated amounts of all other nutrients examined when compared with the inventory. When examined at the group level, the authors also found moderate to strong Pearson’s correlations in the mean amount of nutrients for most of the nutrients assessed (mean *r* = 0.62), but significant differences were observed based on ethnicity and sex for alpha-tocopherol, folate, calcium, fish oil, and garlic for both men and women and vitamin B12 and iron among women (Supplemental Table 1). For reproducibility, Steffen and colleagues (55) statistically compared nutrient intake amounts reported on the two SFQ administrations and determined that intakes for most nutrients remained relatively stable over the 1-year period, regardless of group (i.e., inventory or control); the only statistically significant difference in intakes was noted for vitamin B12 (e.g., 22.9 versus 28.3 μg/day; *p* = 0.0002) (Supplemental Table 2). Thus, the authors concluded that the SFQ is suitable for ranking of participants at the group level but is not adequate for estimating mean nutrient exposures of individuals or groups and that the SFQ does not perform uniformly across ethnic groups but does show similar results at the group level and by sex regardless of the method of collection (55).

### The American Cancer Society’s Cancer Prevention Study-3

Hartman and colleagues (29) examined the relative validity of an FFQ for estimating self-reported DS use with respect to repeat interviewer-administered 24HRs (via telephone) among 684 US adults (age 30–65 years at baseline) who participated in the Cancer Prevention Study-3 (CPS-3). The self-reported FFQ was administered twice, approximately 1 year apart, and probed participants on detailed information about their current use of MVMs and single or combination DSs, such as calcium; folic acid; fish oil; and vitamins C, D, and E (29). Information on the brand, frequency of use, and dosages for each DS consumed was recorded. For the 24HR method, six unannounced, repeat 24HRs were administered via telephone throughout the year on both weekend days and weekdays (29), using the Nutrition Data System for Research (NDSR) Dietary Supplement Assessment Module (DSAM) (http://www.ncc.umn.edu/products/). Participants were prompted to report all DSs consumed in the past 30 days (as opposed to the previous 24 h, which is typically done with foods and beverages), with the assistance of their supplement containers, and to report the exact dose and number of days out of the previous 30 days that the DS was taken (29). The relationships between self-reported supplemental intakes reported on the FFQs and the six 24HRs were assessed via Spearman’s correlation coefficients; specifically, the second FFQ was assessed in relation to (*a*) the average of the six 24HRs and (*b*) the last 24HR completed in closest proximity to the second FFQ (29). For the second FFQ and the average of the 24HRs, correlation coefficients were generally high among men, with the lowest correlation being for calcium (*r* = 0.69), and the highest being for vitamin D (*r* = 0.77); very similar correlations were observed among women (29) (Supplemental Table 1). Correlations between intakes reported on the second FFQ and the last 24HR were slightly different, with correlation coefficients ranging from *r* = 0.65 (fish oil) to *r* = 0.81 (vitamin E) among men and from *r* = 0.59 (fish oil) to *r* = 0.77 (calcium) among women (29) (Supplemental Table 1).

Hartman et al. (29) also sought to assess the reproducibility of the first and second administrations of the FFQ (approximately 1 year apart) for estimating current DS use. For MVMs, substantial agreement between methods was noted, with a weighted κ of 0.67 (29) (Supplemental Table 2). However, for single-nutrient and combination DSs, agreement varied somewhat by DS product type; for example, agreement spanned from moderate (folic acid; κ = 0.47) to substantial (vitamin D; κ = 0.74), with most DSs, on average, exhibiting an agreement of κ = 0.64 (29) (Supplemental Table 2). As a secondary analysis, the investigators also examined the stability (i.e., consistency) of estimates of current DS use reported across three to six 24HRs using ICCs and found that supplement intakes were relatively consistent across recalls, ranging from 0.45 (vitamin C) to 0.84 (calcium) among men and from 0.36 (vitamin D) to 0.84 (calcium) among women (29) (Supplemental Table 2).

## DISCUSSION

DSs are not required to sustain energy needs and are taken voluntarily, often regularly, in discrete doses and by people who tend to have higher educational attainment, healthier diets, and more protective health behaviors—all factors that are associated with higher reporting accuracy (5, 8, 19, 23, 50). Therefore, it should theoretically be much easier to accurately measure DS intake than that of foods and beverages, which rarely possesses these characteristics; yet, little work has been done to demonstrate DS measurement and assessment characteristics in terms of validity and reliability. Only five studies were identified in the literature since 1990 that met the inclusion criteria for this review. All five studies utilized a self-administered questionnaire, either alone or in tandem with another method (e.g., 24HR, DS inventory) (29, 40, 45, 53, 55). A self-administered questionnaire is the most common and cost-effective approach to obtaining information on dietary intake in large-scale research studies, and many of these questionnaires include information on DSs. The findings from the five studies presented in this review suggest that a self-administered questionnaire has adequate reproducibility and reliability, yet only a little over half of questionnaires probe participants on their use of MVM products (49), the most common DS used among the US population (7, 12, 14, 17, 30), and these questionnaires differ markedly in the type of DS information collected (4), making drawing inferences across different studies challenging. A 24HR is advantageous because it can collect rich details on DS use and is typically less cognitively challenging for participants (59); however, there is a large time and cost burden associated with 24HRs, especially when adding DS reporting as an additional module on a recall of food and beverage intakes (59), and 24HRs are generally unable to capture episodically consumed DSs. Therefore, this often renders 24HRs as a suboptimal method for collecting DS use information in most large-scale research settings. An in-person DS product inventory is currently the most intensive method of DS assessment (4). This method is very robust in terms of the accuracy of DS reporting, but not all DS product inventories are conducted in an identical manner. Therefore, while the instrument is sufficient for capturing DS information independently of the approach used, DS inventories may differ in the type and quantity of information collected, depending on the study population they were developed for, and are also very costly to administer, labor intensive, and not commonly conducted outside of NHANES (4, 27). The existing information on the prevalence of DS use and nutrient exposures from DSs measured via a self-administered questionnaire, 24HR, or a DS product inventory (3) and the strengths and limitations of each of these methods are summarized in Table 2. They are also discussed in greater detail elsewhere (4).

No method or recovery biomarker is available to truly validate the self-report of DSs measured via current dietary assessment tools utilized in observational research. Thus, it should be noted that absolute truth cannot be inferred from these studies since each of these DS assessment methods are error prone, and the evaluation only compared error-prone instruments to one another and/or to concentration biomarkers for selected nutrients. In clinical trials testing, adherence to a DS intervention can be evaluated via a 24-h urine collection for some nutrients; however, ensuring the completeness of a urinary collection is critical for understanding the proximity of the estimated value to absolute intakes (58). One method that has been extensively utilized as a measure of completeness for 24-h urine collection is potassium salt para-aminobenzoic acid (PABA) (10, 18, 58); the addition of PABA to an orally administered DS product allows for the measurement of DS product recovery in urine and can therefore aid in monitoring adherence to a DS protocol, given that PABA is thought to have nearly 100% urinary excretion (10, 18, 58). However, this approach is unfortunately not feasible in observational research. In turn, the DS product inventory is considered the gold standard assessment method for observational research (45). An inventory is conducted in person by trained interviewers with participants who show containers for all DS products that they report using. Interviewers record detailed information on each DS such as product name, form, amounts of all ingredients, and the manufacturer. For each DS, follow-up questions address the duration and frequency of use and the dose and can collect additional details, such as motivations for use and if products were recommended by a healthcare provider. In NHANES, product labels are seen for ~90% of DS reports (27). However, supplement inventories are expensive, labor intensive, and infrequently utilized outside of NHANES (4, 27) and the MEC-SURE study (41, 42).

Thus, in the present review, the closest approximation to truth can be inferred from the MEC-SURE study, in which five extensive in-home DS product inventories were conducted by trained interviewers, which included photographs of each DS container, the number of each product reported over time, and detailed information across a year regarding when a product was no longer used or a new product was added (42). Daily, quarterly, and annual nutrient intakes were approximated by summing the nutrient amounts (i.e., frequency and dosage information) across all DSs for each time frame (55). Despite the observational nature of this study, retention across the year was ~95%, and it is one of the few studies to collect DS information from a very diverse population; the questionnaire used was developed based on population-specific content knowledge of the most commonly used products in this cohort, and all nutrient amounts from DSs were estimated via a database specifically created by the researchers and based on the extensive recording of labels specific to each product (64). Thus, the intensive and rigorous MEC-SURE in-home product interview is the closest approximation of truth for DSs to date (25). The results of the MEC-SURE study suggest that the questionnaire performed well for measuring the prevalence of use of DSs (i.e., 74% or more of the products reported by both methods matched) when compared to the in-home product inventory, and the findings across the other studies presented herein support the notion that questionnaires are able to capture simple yes/no information from self-reported assessments. However, other than monitoring trends over time, prevalence of use of DSs alone is not scientifically meaningful to understanding the amount of nutrients and other bioactive food components that humans are exposed to and how this exposure may relate to health outcomes. Therefore, there is a critical need in epidemiological research to improve methods that accurately (or more precisely) quantify nutrients from DSs. The SFQ estimates were significantly different between measures for all nutrients except vitamins A and C, niacin, and calcium, with differences primarily arising from overestimation, depending on the nutrient. Importantly, sex and ethnicity interactions were observed in the misclassification of amounts of nutrients. The fact that specific product information from the SURE database was used and these differences were observed is disconcerting, given that standard practice for the use of DS questionnaire data to obtain estimates on amounts of nutrients relies mainly on default assumptions (49). This limitation was eloquently described by Patterson et al. (45, p. 643) in 1998: “Commonly used epidemiologic methods of assessing supplement use may incorporate significant amounts of error in estimates of some nutrients.” Steffen et al. (55, p. 2486) in 2021 came to a conclusion that was consistent with earlier research: “A self-administered short SFQ can be used in large surveys to identify participants who use 16 categories of dietary supplements at least once a week and can correctly rank participant intakes of nutrients. However, the SFQ does not accurately estimate absolute levels of nutrient intakes from supplements.” These findings are supported by a report from the National Institutes of Health Office of Dietary Supplements that suggests that there is high variability in the number of questions asked about DSs in FFQs, with lists ranging from 3 to 49 different product types (49); even so, questionnaires are the most commonly used method in large epidemiological studies.

The findings from this review suggest that a self-administered questionnaire has adequate reproducibility, especially for DS products that are routinely used. The reproducibility of an instrument can be affected by a number of factors; notably, DS assessment instruments vary in terms of their reference period (short-term versus long-term), as changes over time may influence the consistency in estimates reported on repeat measures (53). In the studies reviewed here, the time that elapsed between questionnaire administrations varied widely, from 3 months to 2.4 years (29, 40, 45, 53, 55). DS usage patterns for certain nutrients can change during the elapsed time period, which may be reflected in a low correlation between the two administrations of a self-administered questionnaire (40). Hartman and colleagues (29) determined that an FFQ exhibited reproducibility over a 1-year period for frequently consumed DSs and that these results were consistent across population subgroups by sex and race and ethnicity. A 24HR may be less likely to capture DSs that are consumed less frequently (i.e., nondaily) than longer-term instruments, but data from the MEC-SURE study suggest that both 1-month (κ = 0.73) and 2-week (κ = 0.75) assessment time frames for recalls were robust with the 1-year time frame (54). However, Cowan et al. (15) noted that the estimated prevalence of overall DS use and the nutrient amounts consumed from DSs differed depending on the DS instrument used in NHANES, with a 30-day reference period capturing a larger proportion of DS users than a 24HR, especially for those consuming more infrequently used products. Thus, findings on reproducibility over time may vary based on cohort, product type, length of reference period, and changes in availability of products, among many other factors.

## SUMMARY AND CONCLUSIONS

DS assessment remains highly inconsistent across different research settings, despite a high prevalence of DS use among the US population (4, 41, 49). An in-person DS product inventory is currently the gold standard method of DS assessment (4). Questionnaires are often utilized because of their low participant burden and cost, but they suffer from lack of detail, require many assumptions to be made, and are limited in scope in terms of the products that are queried (4). A 24HR is traditionally conducted in an open-ended format, capturing specific details on DS products used with relatively low cognitive burden for participants; however, they bypass episodically consumed products and entail a high cost and time commitment (4). All existing methods appear to be adequate in measuring the prevalence and frequency of use at one time point (relative validity) and across time (reproducibility), especially for commonly consumed products, but the amounts of nutrient or other food substance exposures from DSs are most accurately captured from the inventory method.

Regardless of the method used, all existing DS assessment instruments are prone to some degree of largely unquantified measurement error. One limitation of our study is that because the body of research on the validity and reproducibility of DS assessment methods is so small, it is not possible to draw definitive conclusions on the accuracy of supplement assessment instruments in the US at this time. Another limitation of our analysis is that a domestic approach was taken, given the various regulations for DSs around the world. This is reflective of a primary challenge when evaluating DS exposures; presently, there is a lack of international consensus on how best to regulate DSs, and no standardized approaches for ensuring the safety and integrity of DSs exist on a global scale (2). Nevertheless, many challenges observed with DS assessment methodology in the US are paralleled on the global stage (2). Despite these limitations, this study may serve as a foundation for future research seeking to identify the measurement error structure of DS reporting and/or the most comprehensive approach for accurate DS exposure classification and to investigate the validity and reproducibility of DS assessment tools in a variety of research settings and population subgroups and with different nutrients and DS product types.

DSs are widely consumed among the US population (14, 17, 30, 38, 56) and provide significant amounts of nutrients to those who use them (4, 16); quantifying nutrient exposures without the inclusion of exposures from DSs is problematic and may result in inaccurate exposure classification; and the proportion of the population with intakes below recommendations is commonly overestimated, whereas the proportion of the population with intakes above recommendations is typically underestimated (4). This paradigm is particularly important with regard to dietary surveillance; incomplete assessment of nutrient exposures can result in erroneous diet–health and diet–disease associations (25). Consequently, understanding the measurement error structure of DS assessment and its effect on the precision of total micronutrient exposure estimates is of utmost importance, as it directly pertains to the ability to accurately monitor population-level dietary adherence and quantify the relationship between diet and chronic disease risk. More rigorous assessment of the contributions that DSs make toward total exposures to nutrients and other food substances is clearly warranted to improve the accuracy and reproducibility of diet and health research.

## Supplementary Material

Supplementary Material

## Figures and Tables

**Table 1:** Summary of studies evaluating the relative validity and reproducibility of dietary supplement assessment methods

Study	Data collection year	Project name and location	Number of participants and age	Characteristics of participants	DS assessment method	Study objective
Patterson et al. 1998 (45)	1996	A substudy of the Cancer Risk Behavior Survey; Seattle metropolitan area, WA	*n* = 104 (≥21 years, mean age of 44 years)	DS users only; women (57%); mostly non-Hispanic White (91%)	Self-administered supplement questionnaire, in-person interview, and label transcription	Validity question #1: DS questionnaire versus label transcription
						Validity question #2: telephone interview versus label transcription
Satia-Abouta et al. 2003 (53)	2000–2002	A substudy of the Vitamins and Lifestyle (VITAL) study; Seattle metropolitan area, WA	*n* = 220 (50–75 years when recruited)	Equal distributions of sex; mostly non-Hispanic White (94%)	2 self-administered questionnaires and 1 in-home interview with transcriptions of supplement labels	Reproducibility: first questionnaire versus second questionnaire
						Validity question #1: first questionnaire versus in-home interview/transcriptions
						Validity question #2: second questionnaire versus biomarker
Steffen et al. 2021 (55)	2005–2006	The Supplement Reporting (SURE) study; participants from the Multiethnic Cohort, recruited from California and Hawaii	*n* = 1,029 (older adults, mean age of 67.8 years)	Equal distributions of sex; non-Hispanic White, non-Hispanic Black, Hawaiian, Japanese, Latino (US born), Mexican/South American Latino	Short, self-administered SFQ and in-home DS inventory with label transcription	Reproducibility: first SFQ versus second SFQ
						Validity: SFQ versus DS inventory with label transcription
Murphy et al. 2002 (40)	1994–1997	A substudy of the Hawaii–Los Angeles Multiethnic Cohort study	*n* = 2,377 (45–75 years when recruited)	Equal distributions of sex; African American, Japanese American, Latino, Native Hawaiian, and non-Hispanic White	2 DHQs and 3 24HRs	Reproducibility: first DHQ versus second DHQ
						Validity: second DHQ versus 3 24HRs
Hartman et al. 2021 (29)	2015–2016	Cancer Prevention Study-3 Diet Validation substudy; United States, nationwide	*n* = 684 (30–65 years when recruited)	Women (64%); non-Hispanic White (62%), non-Hispanic Black (23%), and Hispanic (15%)	2 FFQs and 6 24HRs	Reproducibility: first FFQ versus second FFQ
						Validity: second FFQ versus 6 24HRs; second FFQ versus the last 24HR closest to the second FFQ

Abbreviations: 24HR, 24-h dietary recall; DHQ, dietary history questionnaire; DS, dietary supplement; FFQ, food frequency questionnaire; SCT, supplement composition table; SFQ, supplement frequency questionnaire.

**Table 2:** Strengths and limitations of each dietary supplement assessment method

	24HR	FFQ	Inventory
Description	Most 24HRs can collect DS use. Some modules facilitate collection and coding for any DS reported but are always used in conjunction with the assessment of foods and beverages.	Most large epidemiological studies use an FFQ to obtain information about diet, and many include DSs. High variability exists in the number of questions asked about DSs, with lists ranging from 3 to 49 different product types (49).	In person, interviewers ask participants to show the containers/labels for the products taken, and they collect detailed information about consumption pattern.
Strengths	A 24HR is an open-ended format in which trained interviewers can capture rich details on DS use, and it does not pose a substantial cognitive burden on the participant.	An FFQ is the most cost-effective approach to obtaining DS information in large-scale research studies.	A DS product inventory is very accurate in terms of DS reporting because detailed information can be collected directly from the DS product label (when available).
Limitations	A standard 24HR that captures intake from midnight to midnight the previous day is unlikely to capture episodically consumed DSs (e.g., a large dose of vitamin D consumed once a week). For ASA24, user error is of concern. It does not perform similarly for certain population groups (i.e., adults aged 40–59 years; African Americans) (44). It requires updated lists of potential DSs to be included and has a limited number of questions incorporated.	FFQs have a high cognitive difficulty (59). A finite list of products can be queried (47). Differences are considerable in the number of products listed, frequency of use responses, duration of use categories, and dosages, making comparisons of intakes across studies difficult (49). Only 56% specifically query the use of MVM products; all query the use of single-vitamin and single-mineral DSs, which are not widely used (49).	The process is costly and time- and labor-intensive. Participants may forget to bring DS containers if inventory is administered outside the home.
	Both 24HRs and FFQs underestimate true dietary intakes (from foods and beverages) as assessed by recovery biomarkers (24, 33, 57). However, recovery biomarkers are only available for energy, protein, sodium, and potassium, all of which are not common in DSs.		
	When detailed information about DSs is not provided, default values are assumed for product brand, formulation, dose, and duration (1, 63). A higher percentage of default values are assigned using the 24HR (26%) when compared to inventory (12%) (43). All methods pose a time burden to participants, adding time to traditional dietary assessment, and pose challenges to capturing complex usage patterns for supplements that are very different from foods (59). All methods are problematic for assessing herbal and other botanical supplements since they rarely provide detail on the product, and all are limited by the quality of existing databases (4).		

Abbreviations: 24HR, 24-h dietary recall; ASA24, Automated Self-Administered 24-h Dietary Assessment Tool; DS, dietary supplement; FFQ, food frequency questionnaire; MVM, multivitamin-mineral.

## SUMMARY POINTS

1. Dietary supplements (DSs) provide a considerable source of nutrients to those that use them, which includes more than half of adults and one-third of children in the United States.
2. DS use substantially changes the proportion of the population at risk of vitamin and mineral inadequacy or excess.
3. Estimations of dietary exposures that do not include nutrients from DSs are incomplete and limited in scientific rigor.
4. A very limited number of studies have been conducted to assess independent methods of DS assessment.
5. Improving measures of dietary exposure will advance our ability to show causal links to health, ultimately enabling successful intervention strategies.
6. In the literature since 1990, we have identified only five relevant studies that compare methods of DS assessment.
7. Without an accurate and reliable assessment tool for DSs, understanding the complex relationships between dietary exposures and health outcomes will continue to be plagued with measurement error.
