# Supplementary material for: Major Gaps in Understanding Dietary Supplement Use in Health and Disease
Source: Annu Rev Nutr. Author manuscript; Available in PMC 2024 May 8. (PMC11078263; doi:10.1146/annurev-nutr-011923-020327)
Supplement: Supplementary Material [file NIHMS1984377-supplement-Supplementary_Material.docx]

**Supplemental Tables**

Supplemental Table 1 Summary of study design and results for studies evaluating the relative validity of dietary supplement assessment methods

| Study | Study design | Main method(s) |  | Reference method(s) | Statistics | Results |
| --- | --- | --- | --- | --- | --- | --- |
| Patterson et al. (1998) | Over the telephone, a vitamin and mineral supplement questionnaire was administered; a self-administered supplement questionnaire was mailed to participants; and at the clinic visit, an in-person interview and label transcription were conducted. | Questionnaire   - Time frame: 1 year - DS category: one-a-day MV (with or without minerals), stress MV, B-complex, or antioxidant mixtures, and single vitamins/minerals for 10 nutrients (calcium; iron; selenium; zinc; folic acid; niacin; and vitamins A, C, D, and E) - Consumption frequency: only for MVs and commonly consumed single vitamins/minerals (calcium, zinc, and vitamins C and E); one per week, 2–6 per week, one per day, and ≥2 per day - Duration: <6 months, ≥ 6 months - Dosage: ranges provided for commonly consumed DSs (calcium, zinc, and vitamins C and E) - Database: for MVs and single supplements missing dose information, default values based on leading brands and informal surveys of supplements available in local drug stores were used - Format: paper-based - Administration: self (by mail) |  | In-person interview/label transcription   - Format: in-person - Administration: interview at a clinic and photocopy of labels - Collected information: self-reported frequency (times per week) of use, duration over the previous year - Database: from photographs - Analysis: the average daily intake was calculated | A kappa statistic–quantified agreement on the types of DSs taken between 2 instruments:  (*a*) DS questionnaire versus in-person interview/label transcription;  (*b*) telephone interview versus in-person interview/label transcription | Questionnaire versus label transcription: Kappa coefficients ranged from 0.36 (calcium) to 0.68 (once-a-day MV) and 0.70 (vitamin E). |
|  |  |  |  |  |  | Telephone interview versus label transcription:  Kappa coefficients ranged from 0.14 (other MV mixtures) to 0.91 (vitamin E) and 0.92 (once-a-day MV). |
|  |  | Telephone interview   - Format: over the telephone - Administration: interview administering a vitamin supplement questionnaire - Collected information: whether participant used a MV or single vitamins over the past year; the type, frequency (times per week), duration (months or years), and dose (open-ended) - Database: for MVs and single supplements with missing dosage information (folic acid, iron, niacin, selenium, and vitamins A and D), default values based on leading brands and informal surveys of supplements available in local drug stores were used - Analysis: the average daily intake was calculated |  |  | Average daily supplemental nutrient intake for 6 nutrients reported on the 2 methods were compared via Spearman’s correlation coefficients. | Questionnaire versus label transcription:  Correlation coefficients ranged from 0.08 (iron) to 0.76 (vitamin C), with a mean of 0.50. Age, gender, and education had no statistically significant effect. |
|  |  |  |  |  |  | Telephone Interview versus label transcription:  Correlation coefficients ranged from 0.27 (iron) to 0.75 (vitamin C), with a mean of 0.50. Age, gender, and education had no statistically significant effect. |
| Satia-Abouta et al. (2003) | A self-administered supplement questionnaire was mailed to participants at baseline and 3 months. At the participants’ home, interview/label transcriptions were conducted and semi-fasting (≥6 hours) blood and spot urine samples were collected. | Questionnaire (see Supplemental Table 2) for current use of DS |  | In-home interview/label transcriptions (i.e., inventory)   - Format: in-person - Administration: home interview (open-ended) and label transcriptions - Collected information: self-reported frequency of use and number of pills taken each time and transcribed nutrient information from each bottle label - Database: for MVs, bottle labels or the *Physicians’ Desk Reference for Nonprescription Drugs and Dietary Supplements* was used. When a participant did not know the brand, content, duration, frequency, or dose, the most common responses were used as default values. - Analysis: the total average daily intake was calculated | Nutrient intakes from DSs among DS users (none and quartiles) reported on both methods were compared by Pearson’s correlation coefficients | Pearson’s correlations were high, with a mean of 0.72; correlations ranged from 0.58 (beta-carotene) to 0.82 (chromium). |
|  |  |  |  | Biomarkers: serum beta-carotene, serum alpha-tocopherol, plasma vitamin C, and urinary calcium (all adjusted for age, sex, race, current smoking, BMI, serum total cholesterol, dietary intake, and/or urinary creatinine) | Nutrient amounts from DSs among DS users (non-users and quartiles of users) reported on the 2 instruments were compared via Pearson’s correlation coefficients | Pearson’s correlations were modest for beta-carotene (0.31) and vitamin C (0.29) and high for alpha-tocopherol (0.69); urinary calcium was not correlated with supplemental calcium intake. |
| Steffen et al. (2021) | Participants were randomly selected and categorized into two groups; the inventory group completed multiple DS assessments over a 1-year period, including an SFQ at baseline and at 1 year, as well as 5 in-home inventories in ~3-month intervals. The control group completed the SFQ at baseline and 1 year later. | Questionnaire (see Supplemental Table 2 for information on the SFQ) |  | In-home DS inventory/label transcriptions   - Format: in-person - Administration: home interview (open-ended) and label transcriptions by trained interviewers conducted at baseline and every 3 months for 1 year - Collected information: self-reported DS information on dose, frequency, and number of pills consumed. Product containers were transcribed for each DS reported, and any DS label that was not already in the SCT was photographed. - Database: University of Hawaii Cancer Center Supplement Composition Table - Analysis: the nutrient contents entered into the SCT were then multiplied by the dose consumed during each 3-month interval to estimate nutrient amounts per DS. Daily, quarterly, and annual amounts consumed were calculated by summing the nutrient amounts across all DSs for each time frame. | Percent agreement for the proportion of the population reporting weekly DS use on the SFQ and the in-home inventory methods by DS product type, based on classification matches | Percent agreement between the SFQ and the in-home inventory ranged from 88% (antioxidants) to 97% (selenium) for nearly all DS, except vitamin D (74%). |
|  |  |  |  |  | Comparison of nutrient intake amounts from DSs between the in-home inventory and SFQ via a paired t-test and via Pearson’s correlation coefficients | Pearson’s correlations of nutrient intake amounts from DS between the two methods predominantly varied from low (*r* = 0.48; vitamin B6) to high (*r* = 0.75; folate), apart from iron (*r* = 0.29), which was considered negligible. Mean correlation was *r* = 0.62 across 17 nutrients. |
| Murphy et al. (2002) | The first DHQ was completed by participants at study initiation. 3 24HRs were then collected in approximately 1-month intervals. After all the 24HRs were collected, the second DHQ was collected ~4–6 weeks later. | Questionnaire (see [Supplemental Table 2](#tb3) for information on the DHQ) |  | 24HR   - Format: over telephone - Administration: interview (only on the days the participants considered to be typical of their usual intake; days of the week were randomly assigned to obtain a balance of all 7 days) - Collected information: whether participants used any DSs, the type and the brand name of the DSs, place of purchase, dosage, number of tablets per dose, and the number of tablets taken - Database: Cancer Research Center of Hawaii’s Supplement Composition Table. When a participant did not know the dose, default values based on the most commonly consumed product in the same category were used. - Analysis: the average of 3 24HRs was calculated | Frequency of use in 2 categories (never and ever use) via kappa statistics | Kappa statistics ranged from 0.17 (vitamin A) to 0.72 (vitamin E). |
|  |  |  |  |  | Frequency of use in 6 categories (never to ≥3 tablets per day) via weighted kappa statistics | Weighted kappa statistics ranged from 0.16 (vitamin A) to 0.74 (vitamin E).  Agreement was somewhat lower for African American (κ = 0.43) and Latino (κ = 0.41) subjects than for Japanese American, Hawaiian, and non-Hispanic White subjects (all κ ≥ 0.52) |
|  |  |  |  |  | Nutrient intake estimates among DS users reported via the 2 methods were compared using Pearson’s correlation coefficients | Pearson’s correlation coefficients ranged from 0.11 (folate) to 0.71 (vitamin E). |
| Hartman et al. (2021) | The self-reported FFQ was administered twice, approximately 1 year apart. Over the year, 6 repeat interviewer-administered 24HRs were collected. | Questionnaire (see [Supplemental Table 2](#tb3) for information on the FFQ) |  | 24HR (using the NDSR Dietary Supplement Assessment Module that queries 30-day supplement intake history)   - Format: via telephone - Administration: unannounced interview (4 on weekdays and 2 on weekend days) - Collected information: self-reported any DS consumed, frequency of use and dose - Database: NDSR database - Analysis: the total average daily intake was calculated | Supplemental nutrient intakes reported on the FFQs and 6 24HRs were compared via Spearman’s correlation coefficients | Second FFQ versus 6 24HR:  Spearman’s correlations ranged from 0.65 (fish oil for women) to 0.77 (vitamin D for men, calcium for women); results were comparable by sex  Second FFQ versus the last 24HR closest to the second FFQ:  Spearman’s correlations varied from *r* = 0.65 (fish oil) to *r* = 0.81 (vitamin E) among men, and from *r* = 0.59 (fish oil) to *r* = 0.77 (calcium) among women. |

Abbreviations: 24HR, 24-h dietary recall; DHQ, dietary history questionnaire; DS, dietary supplement; FFQ, food frequency questionnaire; MV, multivitamin; NDSR, Nutrition Data System for Research; SCT, Supplement Composition Table; SFQ, supplement frequency questionnaire.

Supplemental Table 2 Summary of study design and results for studies evaluating the reproducibility of dietary supplement assessment methods

| Study | Study design | DS assessment method | Analysis | Results |
| --- | --- | --- | --- | --- |
| Satia-Abouta et al. (2003) | A self-administered supplement questionnaire was mailed to participants at baseline and 3 months | Questionnaire   - Time frame: past use over the previous10 years (reproducibility) and current use (validity) - DS category: MVs and 10 vitamins and 6 minerals from all other types - Consumption frequency: 1–2 days per week, 3–4 days per week, 5–6 days per week, 7 days per week. - Duration over the previous 10 years: 1–3 years, 4–6 years, 7–9 years, ≥10 years - Dosage: participants chose from the options on usual dose per day based on the most common formulations - Database: for MVs, bottle labels or the *Physicians’ Desk Reference for Nonprescription Drugs and Dietary Supplements* was used. For MVs used currently, participants could choose from a list of 16 brand names or provide information. For MVs used in the past, participants could choose from a list of brand names reflecting past market availability or answer “don’t know.” When a participant did not know the brand, content, duration, frequency, or dose, the most common responses were used as default. - Format: paper-based - Administration: self-administered (by mail) | The distribution of supplemental nutrient intake (none and quartiles) by weighted kappa coefficients | Weighted kappa statistics ranged from 0.58 (calcium) to 0.78 (multivitamins), with mean of 0.69. |
|  |  |  | Log-transformed supplemental nutrient intake reported in both FFQ administrations were compared using ICCs | Mean ICCs of 0.79. Correlations ranged from 0.69 (beta-carotene) to 0.87 (vitamin E). |
| Steffen et al. (2021) | 2 SFQs in a 1-year interval | SFQ   - Time frame: weekly use of DSs over the previous year - DS category: MVMs (one-a-day, B-complex, antioxidant types); vitamins A, C, D, E, and B-12; and beta-carotene, folic acid, calcium, selenium, iron, zinc, fish oil/omega-3s, and garlic DS. - Consumption frequency: 1–3 times/week, 4–6 times/week, and once per day - Duration of use: ≤1 year, 2–4 years, 5–9 years, or ≥10 years - Dosage: dosages were obtained for vitamins C and E, each with four choices for the dose. Default doses from the SCT were used for all other nutrients. - Database: University of Hawaii Cancer Center SCT. - Format: paper-based - Administration: self-administered during the in-home visit (inventory group) or via mail (control group) | Geometric means of supplemental nutrient intakes reported in the SFQ at baseline and at 1 year were calculated. Differences in log-transformed supplemental nutrient intakes reported in both SFQ administrations were calculated. | Supplemental nutrient intake remained consistent from the baseline to follow-up administrations of the SFQ for most nutrients, with the majority of mean differences <0.1 SD of the log-transformed nutrients, except for vitamin B12 intake. |
| Murphy et al. (2002) | The first DHQ was completed by participants at study initiation via postal mail. The second DHQ was collected, on average, 2.4 years later via mail. | DHQ   - Time frame: any DS used for at least 1 week during the past year - DS category: MV or MVM; vitamins A, C, and E; β-carotene; calcium; selenium; and iron - Consumption frequency: never, 1–3 tablets per week, 4–6 tablets per week, 1 tablet per day, 2 tablets per day, ≥3 tablets per day - Dosage: participants chose the approximate dosage per tablet from several dose ranges - Database: for MVs or MVMs, composites of two most commonly reported brands from the dietary recalls were used. When a participant did not know the dose, the lowest amount category was assumed. - Format: paper-based - Administration: self-administered (by mail) | Frequency of use in 2 categories (never and ever use) by kappa statistics | Kappa statistics ranged from 0.39 (vitamin A) to 0.64 (vitamin C). |
|  |  |  | Frequency of use in 6 categories (never to ≥3 tablets per day) by weighted kappa statistics | Weighted kappa statistics ranged from 0.39 (vitamin A) to 0.64 (vitamin C). |
| Hartman et al. (2021) | 2 FFQs in 1 year interval | FFQ   - Time frame: current use - DS category: MVM and 7 vitamins and minerals - Consumption frequency: ranges provided to the participant - Dosage: participants chose the approximate dosage per tablet from several dose ranges - Database: information from brand-name options and a write-in option - Format: paper-based - Administration: self-administered (by mail) | Ranked nutrient intake estimates between FFQ administrations by Spearman’s correlation coefficients | Spearman’s correlation coefficients ranged from 0.57 (vitamin E, women) to 0.70 (vitamins C and D, men). Correlations were similar between men and women but tended to be lower for NH Black participants. |
|  |  |  | Comparison of ordinal categorical responses reported on the two FFQs by weighted kappa statistics | The weighted kappa for MVMs in the overall population was 0.67, with a tendency for lower agreement for NH Black participants. |
|  |  |  | ICCs were used to examine the consistency of estimates of DS use across 3 to 6 24HRs | DS use was generally consistent; ICCs ranged from 0.45 (vitamin C) to 0.84 (calcium) among men and from 0.36 (vitamin D) to 0.84 (calcium) among women. |

Abbreviations: DHQ, dietary history questionnaire; DS, dietary supplement; FFQ, food frequency questionnaire; ICC, intraclass correlation coefficient; NH, non-Hispanic; MV, multivitamin; MVM, multivitamin-mineral; SCT, Supplement Composition Table; SD, standard deviation; SFQ, supplement frequency questionnaire.
